# Intelligent Framework for Adverse Drug Event Identification Using Large Language Models and Retrieval-Augmented Generation: Development and Evaluation Study

**DOI:** 10.2196/84086

**Published:** 2026-08-10

**Authors:** Junlong Ma, Xuehong Wu, Zeying Feng, Yun Kuang, Zhendong Ding, Min Li, Guoping Yang

**Affiliations:** 1 Department of Pharmacy, Xiangya Hospital, Central South University, Changsha, Hunan, China, 1 0731 88618933; 2 School of Computer Science and Engineering, Central South University, Changsha, Hunan, China; 3 Clinical Trial Institution Office, Liuzhou Hospital of Guangzhou Women and Children's Medical Center, Liuzhou, Guangxi, China; 4 Center of Clinical Pharmacology, The Third Xiangya Hospital, Central South University, No 138 Tongzipo Road, Yuelu District,Changsha, Hunan, 410013, China, 86 0731 88618933; 5 Department of Anesthesiology, The Third Xiangya Hospital, Central South University, Changsha, Hunan, China; 6 Xiangya School of Pharmaceutical Sciences, Central South University, Changsha, Hunan, China

**Keywords:** adverse drug event, large language model, retrieval-augmented generation, Chinese clinical notes, pharmacovigilance

## Abstract

**Background:**

Adverse drug events (ADEs) pose significant public health challenges and economic burdens. While substantial ADE information is documented in unstructured clinical notes, its extraction remains difficult due to semantic complexity. Large language models (LLMs) offer promising text comprehension capabilities but are often hindered by domain-specific hallucinations.

**Objective:**

This study aims to evaluate the effectiveness of retrieval-augmented generation (RAG) in improving the identification of ADEs using LLMs from Chinese clinical narratives and to establish a paradigm for this task.

**Methods:**

We collected and preprocessed 19,983 Chinese clinical notes, retaining 18,432 high-quality records. Following a rigorous annotation and deduplication process, we established a gold-standard reference dataset (n=2510) and an ADE knowledge base (n=5144) using a standardized JSON schema. We evaluated 3 state-of-the-art LLMs (DeepSeek-V3 [DeepSeek], ERNIE 3.5-8K [Baidu], and GPT-4o [OpenAI]) under 3 prompt strategies: nonaugmented generation (NAG), static-augmented generation (SAG), and RAG. Performance was comprehensively assessed using precision, recall, and *F*_1_-score across 3 recognition matching levels (L1 exact, L2 sentence, and L3 overlap) via 1000 bootstrap resamples. Model robustness was further validated from real-world clinical progress notes, reflecting real-world ADE prevalence.

**Results:**

We successfully constructed and publicly released the first Chinese ADE corpus derived from clinical notes. Across the tested LLMs, RAG yielded higher *F*_1_-scores than NAG and SAG at the L3 level. The optimal configuration, DeepSeek-V3 with RAG, achieved an overall L3-level *F*_1_-score of 0.9638 (95% CI 0.9541‐0.9727). Notably, the RAG approach increased the recall of GPT-4o from 0.6419 under NAG to 0.9241 under RAG (FDR *P*=.003). Evaluation on real-world datasets demonstrated clinical utility, with the RAG prompt maintaining high discriminatory capability (specificity: 0.9821; *F*_2_-score: 0.8885). Error analysis revealed that RAG successfully resolved common identification errors, both omissions and commissions, that were intractable for nonaugmented models.

**Conclusions:**

Synergizing a curated domain-specific knowledge base with LLMs via a RAG architecture is an effective strategy for accurately identifying ADEs in unstructured Chinese clinical notes. This approach can mitigate hallucinations in LLMs, providing a foundational open-source benchmark and a robust technical framework to advance pharmacovigilance, drug safety research, and clinical decision support.

## Introduction

Adverse drug events (ADEs) are defined as harmful reactions that are unrelated to the intended therapeutic effects of medications when they are administered at standard dosages and according to standard regimens. ADEs constitute a significant global public health concern. They are among the main causes of hospitalization and mortality in both developed and developing countries [1]. Meta-analyses reveal that approximately 5%‐10% of patients in health care institutions experience ADEs [2]. Furthermore, the economic burden attributable to ADEs within health care systems worldwide exceeds US $42 billion annually [3]. The precise identification and comprehensive evaluation of ADEs, including elucidating their underlying etiologies, are imperative for mitigating harm and augmenting the quality of clinical care [4]. Consequently, the surveillance and management of ADEs have emerged as critical public health priorities, wherein the accurate extraction of ADE-related information can significantly enhance drug safety and foster rational pharmacotherapy.

A substantial proportion of ADE information remains embedded within unstructured, narrative clinical notes, presenting formidable challenges to conventional manual review and extraction methodologies, which are often inefficient and labor-intensive [5,6]. The advent of large language model (LLM)–based tools, such as ChatGPT, offers a promising way to efficiently and accessibly identify and retrieve ADE information [7]. Trained on vast corpora of textual data, LLMs demonstrate remarkable abilities in cross-domain text comprehension, logical inference, and human-like natural language generation [8]. Empirical studies have demonstrated their utility across diverse medical applications, including disease diagnosis and management [9], patient education and counseling [10], clinical text analysis [11], and postoperative risk stratification [12]. Despite growing evidence attesting to the value of LLMs in multiple medical domains, their potential in pharmacovigilance, particularly in the detection of ADEs in clinical notes, remains insufficiently explored. Furthermore, as LLMs are primarily pretrained on publicly available datasets without domain-specific clinical fine-tuning, they are prone to generating “hallucinations,” a phenomenon warranting heightened vigilance in health care contexts [13,14]. For instance, Williams et al [15] found that GPT-4 and GPT-3.5-turbo models produced fabricated patient visit summaries at an alarming rate of up to 42%.

Retrieval-augmented generation (RAG) architectures enhance the performance of LLMs by integrating external information retrieval mechanisms, thus improving response accuracy and practical applicability [16]. By dynamically combining domain-specific knowledge bases with user queries, RAG provides comprehensive, high-quality contextual information, reducing the likelihood of erroneous model outputs and effectively addressing the hallucination challenge [17,18]. Additionally, RAG affords access to the latest reliable knowledge without the substantial costs of extensive model fine-tuning [19]. Recent studies have highlighted RAG’s superiority over standard LLMs in biomedical tasks, including question answering, text and image generation, and clinical scenario interpretation [20]. For instance, Li et al [21] showed that combining RAG with LLMs substantially improves the accuracy and reliability of COVID-19 fact-checking, successfully overcoming the inherent hallucination and context-inaccuracy issues [21]. Nonetheless, the efficacy of RAG depends on the availability of trustworthy, unbiased data, and the quality of external domain knowledge significantly affects performance [22]. Currently, ADE-related knowledge is fragmented and lacks structured representation, posing significant barriers to the deployment of RAG and LLM frameworks for ADE extraction. Therefore, there is a compelling need to construct a high-quality, structured Chinese ADE corpus.

This study aims to evaluate the effectiveness of RAG in enhancing LLM-based identification of ADE information within Chinese clinical notes. By integrating a curated knowledge base and using several foundational LLMs, this research systematically assesses multiple RAG-based architectures. Through comparative analysis of model performance under various configurations, this study seeks to establish an optimal paradigm for extracting ADE information from clinical notes, thereby providing robust technical support for pharmacovigilance and rational pharmacotherapy.

## Methods

### Datasets and Data Preprocessing

**Figure 1.**
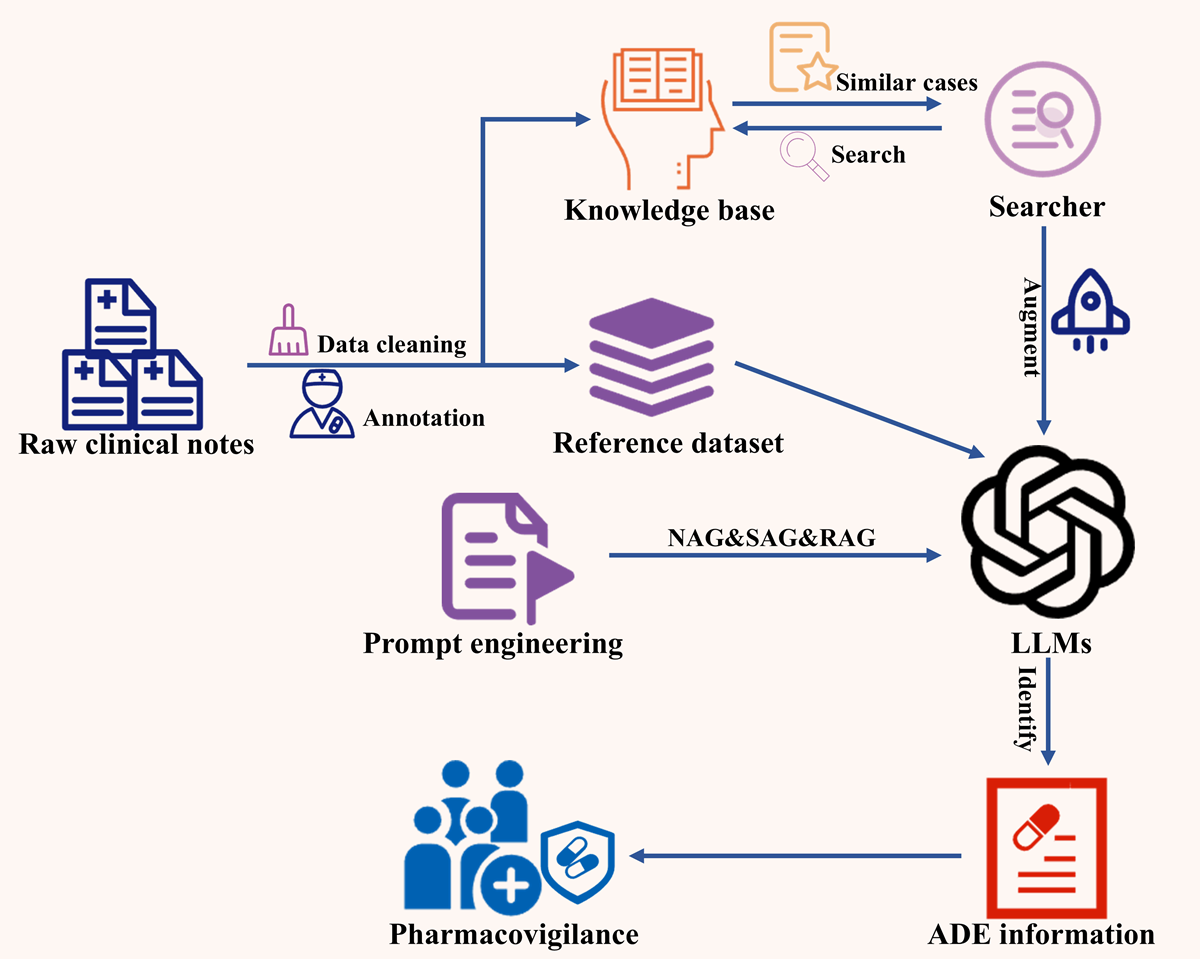
Workflow of the adverse drug event recognition study. ADE: adverse drug event; LLM: large language model; NAG: nonaugmented generation; RAG: retrieval-augmented generation; SAG: static-augmented generation.

The study flow is illustrated in Figure 1. A total of 19,983 clinical notes were randomly collected from multiple large-scale tertiary hospitals, encompassing records from the years 2007 through 2023. These records covered a variety of document types, including ward round notes, initial clinical summaries, transfusion logs, handover reports, and routine progress documentation. A rigorous data preprocessing pipeline was implemented to protect patient confidentiality and improve data integrity. During the deidentification phase, all personally identifiable information relating to patients and physicians was uniformly replaced with generic terms, such as “Patient” and “Physician.” Similarly, health care institution names were anonymized.

To ensure data quality, a multitiered filtration protocol was implemented: (1) removal of records manifesting abnormal, nonsensical, or noncompliant content with established medical documentation standards; (2) exclusion of entries shorter than 100 characters; and (3) elimination of excessively long records exceeding 3000 characters that contained abundant irrelevant content or extraneous tags. Following this systematic preprocessing, 18,450 records meeting the quality criteria were initially retained. To prevent potential patient-level data leakage, we further verified all records using a double-deidentified patient master index. This verification showed that the 18,450 notes corresponded to 18,432 unique patients, with only 14 patients contributing multiple notes. After retaining 1 note per patient and removing 18 duplicate-patient records, all of which were ADE-negative, 18,432 patient-level clinical records were retained.

### Dataset Partitioning and Annotation Generation

The curated dataset was randomly partitioned into 2 distinct subsets with a 3:7 ratio: (1) a standard reference dataset comprising 5535 entries, designated as the gold standard to rigorously validate the efficacy of ADE identification methodologies; (2) an ADE case knowledge base consisting of 12,897 entries, intended for providing domain-specific knowledge. Both datasets were processed using a standardized annotation protocol. To eliminate potential automation bias, initial ADE preannotations were generated using Qwen-turbo (model version updated April 28, 2025; temperature=1.0, top_*P*=.9, penalty_score=1.05), a model explicitly distinct from the 3 evaluated LLMs. These preannotations were subsequently reviewed and revised by clinical pharmacists with 2‐10 years of pharmacovigilance experience. All annotators were fully blinded to the preannotation model and the evaluation design, receiving only raw clinical notes and machine-generated labels. Interrater reliability was assessed using Cohen κ based on 5535 clinical notes independently reviewed by 2 annotators, yielding a high agreement (Cohen κ=0.86). Any disagreements between annotators were resolved through consensus discussion, thereby confirming the high consistency and robustness of the gold-standard reference dataset.

During the annotation and review process, we observed a high degree of homogeneity in both clinical notes and the ADE annotations within the positive samples. Such redundancy has the potential to introduce bias in subsequent analyses, particularly as cases sharing identical ADE categories may collectively succeed or fail in recognition, thereby compromising the robustness of evaluative conclusions. To address this issue, we implemented a strict deduplication and balancing procedure to derive the final datasets. First, within the initial standard reference dataset of 5535 entries (comprising 2789 positive and 2746 negative instances), we deduplicated the positive reference set by limiting each drug entity to no more than 3 appearances. Deduplication was performed strictly according to the annotated drug entity name, without collapsing specific drugs into a generic category. Following deduplication, the positive reference set was refined to 1228 records. Correspondingly, an equivalent sample of 1282 instances was randomly selected from the 2746 negative cases, resulting in a balanced, final standard reference dataset of 2510 records. Subsequently, a similar deduplication and balancing process was applied to the initial dataset comprising 12,897 clinical records. This resulted in the creation of a final ADE knowledge base comprising 5144 records (2273 positive cases and 2871 negative cases).

To standardize the annotation process and accurately capture the subtle characteristics of ADE descriptions in clinical notes, a JSON schema was adopted for ADE annotation and recognition tasks (Figure S1 in Multimedia Appendix 1). Specifically, the JSON schema encapsulates a structured array wherein each ADE incident is described as an object comprising three pivotal fields: (1) sentence: the original textual fragment characterizing the ADE event; (2) drugs: an array listing the implicated pharmaceutical agents, accommodating polypharmacy interactions; and (3) reactions: an array detailing the corresponding ADE entities, supporting multiple concurrent reactions. If no ADE is identified within a record, an empty array “[]” is returned.

Using a JSON-based model for ADE information has several distinct advantages: (1) structured storage facilitates efficient data organization through standardized fields, thereby providing a solid foundation for downstream tasks such as causal inference and association analysis; (2) stringent field constraints (eg, requiring nonempty reaction arrays) enforce rigorous accuracy requirements for ADE entity recognition; and (3) compatibility with LLM input-output paradigms enhances the efficacy of generative models in extracting and completing ADE-related information.

### Model Setting and Prompt Design

**Figure 2.**
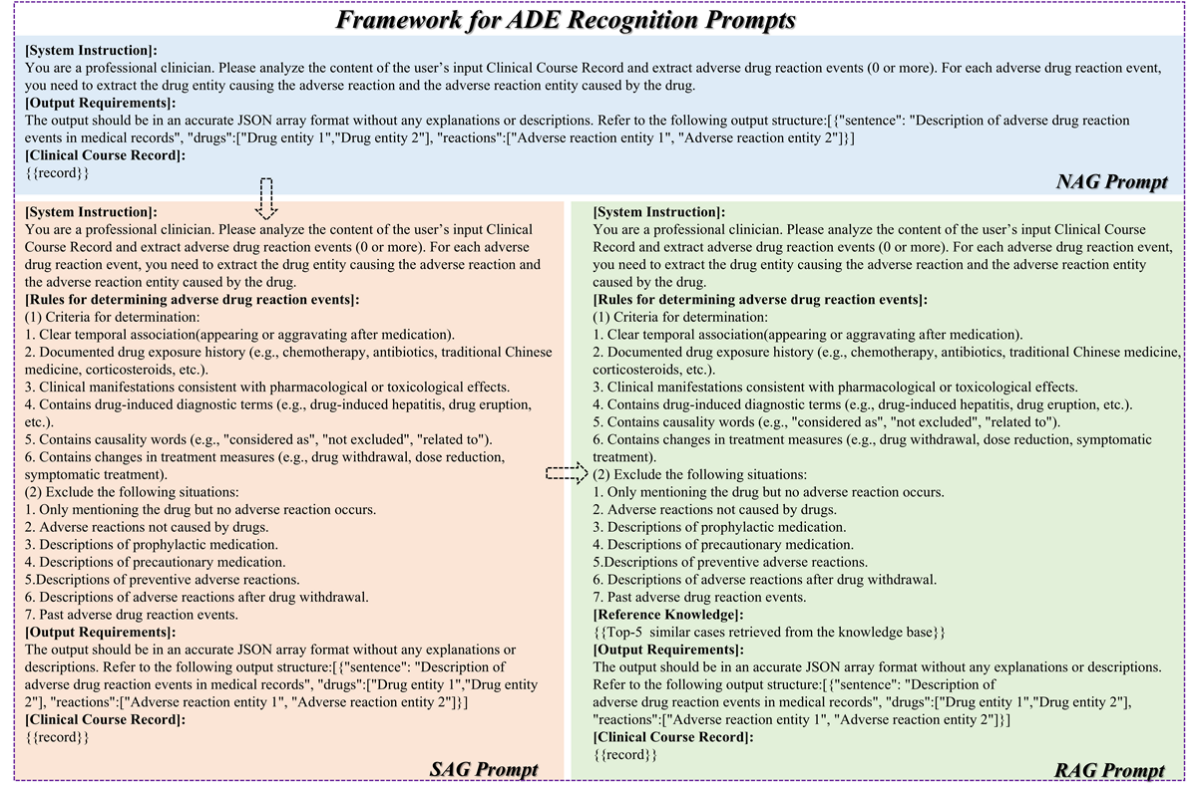
Prompt engineering framework for ADE recognition, translated into English for reader comprehension. The schematic details 3 step-wise augmentation strategies for large language models. (A) Nonaugmented generation serving as a zero-shot baseline. (B) Static-augmented generation integrating static clinical diagnostic rules. (C) Retrieval-augmented generation dynamically injecting top 5 similar cases for context-aware, few-shot prompting. ADE: adverse drug event; LLM: large language model; NAG: nonaugmented generation; RAG: retrieval-augmented generation; SAG: static-augmented generation.

The study used 3 state-of-the-art LLMs: DeepSeek-V3 (DeepSeek), ERNIE 3.5-8K (Baidu), and GPT-4o (OpenAI). Specifically, DeepSeek-V3 offers cutting-edge open-source performance with privacy-preserving local deployment; ERNIE 3.5-8K provides a commercial baseline optimized for Chinese clinical natural language processing; and GPT-4o serves as a top-tier, general-purpose international benchmark. Together, they represent a broad spectrum of open-source versus closed-source, Chinese-specialized versus general-purpose, and local versus cloud paradigms. The key to leveraging these LLMs was prompt engineering. To systematically assess the impact of prompt design and RAG strategy on ADE recognition performance, a progressive optimization framework was implemented: (1) nonaugmented generation (NAG)—a zero-shot prompting baseline strategy supplying the LLM with ADE identification instructions without any supplementary knowledge, thereby delineating the model’s innate capabilities; (2) static-augmented generation (SAG)—building upon NAG, this zero-shot approach incorporates static clinical knowledge descriptions of ADE concepts to formulate semantically constrained recognition prompts, evaluating the influence of conceptual knowledge injection; and (3) RAG—further advancing SAG into a dynamic few-shot prompting strategy with a dynamic retrieval mechanism that fetches analogous cases from the ADE knowledge base in real time, yielding context-aware prompts optimized for the complexity of clinical notes (Figure 2 and Figure S3 in Multimedia Appendix 1).

According to the retrieval schema of the RAG framework proposed in this paper, each case is structured as a key-value pair: the key is the original ADE text snippet, and the value is a standardized JSON string. Keys were vectorized using the shaw/dmeta-embedding-zh model (vector dimension=768) and indexed in a Milvus (v2.5.2; Zilliz) database, with corresponding JSONs stored as structured metadata. During dynamic retrieval, input clinical notes are split into sentences using punctuation marks and processed via a sliding window (size=3, step=2). These contextual segments are vectorized to retrieve the top 5 most similar ADE entries from the Milvus database via cosine similarity. The retrieval module achieved a Recall@5 of 0.908 and a mean reciprocal rank of 0.874, empirically verifying its ability to accurately fetch relevant ADE cases and reduce model hallucinations.

This prompt architecture follows a logical progression: from baseline capability verification to knowledge-enhanced refinement and adaptive contextualization, establishing a rigorous and reusable experimental paradigm for quantitatively evaluating knowledge augmentation strategies. All LLM inference, prompting, and ADE extraction tasks were conducted natively in Chinese. The English prompt text and translations shown in figures and tables are provided solely for reader comprehension and were not used as model inputs. The NAG instruction comprises 3 sections: “System Instruction,” “Output Requirement,” and “Clinical Course Record.” The “System Instruction” guides ADE identification, while the “Output Requirement” specifies adherence to the ADE JSON schema. The “Clinical Course Record” represents the data to be analyzed, with “record” acting as a placeholder for later use. The SAG instruction mirrors NAG but elaborates on ADE criteria, including temporal associations, drug exposure history, clinical manifestations, and causality terms. Exclusion criteria are also defined, including mentions of drugs without adverse reactions, non–drug-related reactions, prophylactic medications, vigilant use, postdiscontinuation reactions, and prior ADEs. The RAG instruction extends SAG by adding a “Reference Knowledge” section. Specifically, the retrieved top 5 ADE reference cases are explicitly injected into this section of the structured RAG prompt template (Figure 2), providing contextual domain knowledge to enhance the LLMs’ accurate and interpretable ADE identification.

### Experimental Setup

Our entire experimental pipeline was implemented natively in Java (JDK 1.8; Oracle Corporation), using the HttpClient library to establish seamless communication with cloud-based model services. Although DeepSeek-V3 permits privacy-preserving local deployment, the present experiments used its cloud API version, together with the cloud APIs of ERNIE 3.5-8K and GPT-4o, to ensure consistent batch inference and experimental efficiency. All API calls used deidentified Chinese clinical-note inputs and Chinese prompt templates. To guarantee complete study reproducibility, the exact versions and configurations of the invoked models are specified as follows: DeepSeek-V3 (updated March 24, 2025; temperature=0.3, top_*P*=.70, penalty_score=1.0), ERNIE 3.5-8K (updated December 22, 2024; temperature=0.3, top_*P*=.70, penalty_score=1.0), and GPT-4o (updated November 20, 2024; temperature=0.3, top_*P*=.70, frequency_penalty=0, presence_penalty=0).

### Statistical Metrics for Model Evaluation

The experimental outcomes were evaluated using precision, recall, the *F*_1_-score, specificity, the *F*_2_-score, G-mean, and the Cohen κ metric. Within the reference dataset, clinical notes containing ADEs were designated as positive instances, whereas those devoid of ADEs were classified as negative instances. The metrics were computed as follows:


$$\text{Precision = }\frac{\text{TP }}{\text{TP+FP}}$$

$$\text{Recall = }\frac{\text{TP}}{\text{TP+FN}}$$

$$F_{1}\text{-score}=\frac{\text{2×Precision×Recall}}{\text{Precision + Recall}}$$

$$F_{2}\text{-score}=\frac{5\times \text{Precision}\times \text{Recall}}{4\times \text{Precision}+\text{Recall}}$$

$$Specificity=\frac{TN}{TN+FP}$$

$$G\_Mean=\sqrt{Recall*Specificity}$$

$$P_{o}=\frac{TP+TN}{N}$$

$$P_{e}=\frac{\left(TP+FN)*(TP+FP)+(FP+TN)*(FN+TN\right)}{N^{2}}$$

$$K=\frac{P_{o}-P_{e}}{1-P_{e}}$$

TP denotes the count of true positives where the model correctly identifies an instance as positive; FP represents false positives where the model identifies an instance as positive but the reference set labels it as negative; FN signifies false negatives where the model fails to identify an instance marked positive in the reference set. P_o_ represents the actual observed accuracy and P_e_ represents the chance-expected accuracy. All reported metrics were computed at the document level using a clinical note, rather than an individual ADE entity, as the unit of evaluation.

It is important to recognize that identifying ADEs is a complex task. For any given sample in the standard reference set, a more nuanced determination of recognition accuracy is required. To this end, we have defined 3 levels of recognition precision: L1, L2, and L3 (Table 1). The L1, L2, and L3 rules were used to determine TP versus FN status only among ADE-positive notes. L1 (exact match) strictly requires complete alignment of the predicted sentence, drug array, and adverse reaction array with the gold standard. Under L1, partial matches (eg, correct drug but incomplete reactions) were rejected as TP and counted as FN at the document level. Conversely, L2 (sentence-level match) counted a note as TP when the predicted and gold-standard sentences aligned, even if the drug or reaction entities were incomplete or partially discrepant. L3 (overlap match) further relaxes this criterion, counting a TP if at least one drug or adverse reaction entity overlaps, regardless of overall array completeness. Figure S4 in Multimedia Appendix 1 provides illustrative examples to further elucidate these definitions. Paired bootstrap tests were applied to the 1000 bootstrap resamples to evaluate the statistical significance of performance differences across models, prompts, and matching levels. *P* values were adjusted using the Benjamini-Hochberg false discovery rate (FDR) procedure, and statistical significance was defined as FDR *P*<.05.

To evaluate model robustness under real-world clinical conditions, we additionally assessed DeepSeek-V3 using uncurated clinical progress notes sampled from the original pool of 19,983 raw records. Prior to calculating the metrics, the remaining records that were excluded from the curated dataset were also annotated with supplementary ADE labels using the same JSON schema and a consistent manual review protocol. In total, 1000 resampling iterations were performed, with 1000 notes randomly selected in each iteration and evaluated under the NAG, SAG, and RAG prompting strategies. The average ADE-positive prevalence was approximately 4.6%. Given this imbalance, specificity, *F*_2_-score, G-mean, and Cohen κ were calculated in addition to precision, recall, and *F*_1_-score to assess false-positive control, recall-oriented detection, overall discrimination, and agreement with the gold standard. To further assess performance on challenging clinical language, we constructed a targeted 100-note subset containing ADE negation, abbreviations, temporal and causal contexts, and evaluated it using DeepSeek-V3 with RAG. All reported metrics, including precision, recall, *F*_1_-score, specificity, *F*_2_-score, G-mean, and Cohen κ, were computed at the document level using a clinical note.

Statistical analyses were performed in Python (version 3.10; Python Software Foundation) using *NumPy*, *pandas*, *SciPy*, and *scikit-learn* packages for bootstrap resampling, percentile-based 95% CIs, paired bootstrap tests, Cohen κ, and classification metrics.

**Table 1.** Definition of identification precision level^a^.

Precision level	Description
L1	The identified ADEs^b^ are completely consistent with the annotated ADEs, including the number of identified ADEs, as well as the sentences, drugs, and reactions within each ADE.
L2	The identified ADEs are roughly consistent with the annotated ADEs. The number of ADEs matches, and the sentences within the ADEs are the same, but there are discrepancies in the drug and reaction entities.
L3	The number of identified ADEs does not match the number of annotated ADEs, but there is an overlap between them.

a L1 (exact match of sentences, drugs, and reactions), L2 (sentence-level match with drug or reaction discrepancies), and L3 (partial overlap of entities).

b ADE: adverse drug event.

### Ethical Considerations

This retrospective study was approved by the Ethics Review Board of Xiangya Hospital of Central South University (2025091288). The requirement for informed consent was waived because this study involved a secondary analysis of existing clinical notes. All data used were anonymized to ensure participant privacy. No personally identifiable information was included in the study, nor was any such information disclosed to any LLMs.

## Results

### Construction Results of the Standard Reference Set and ADE Knowledge Base

**Figure 3.**
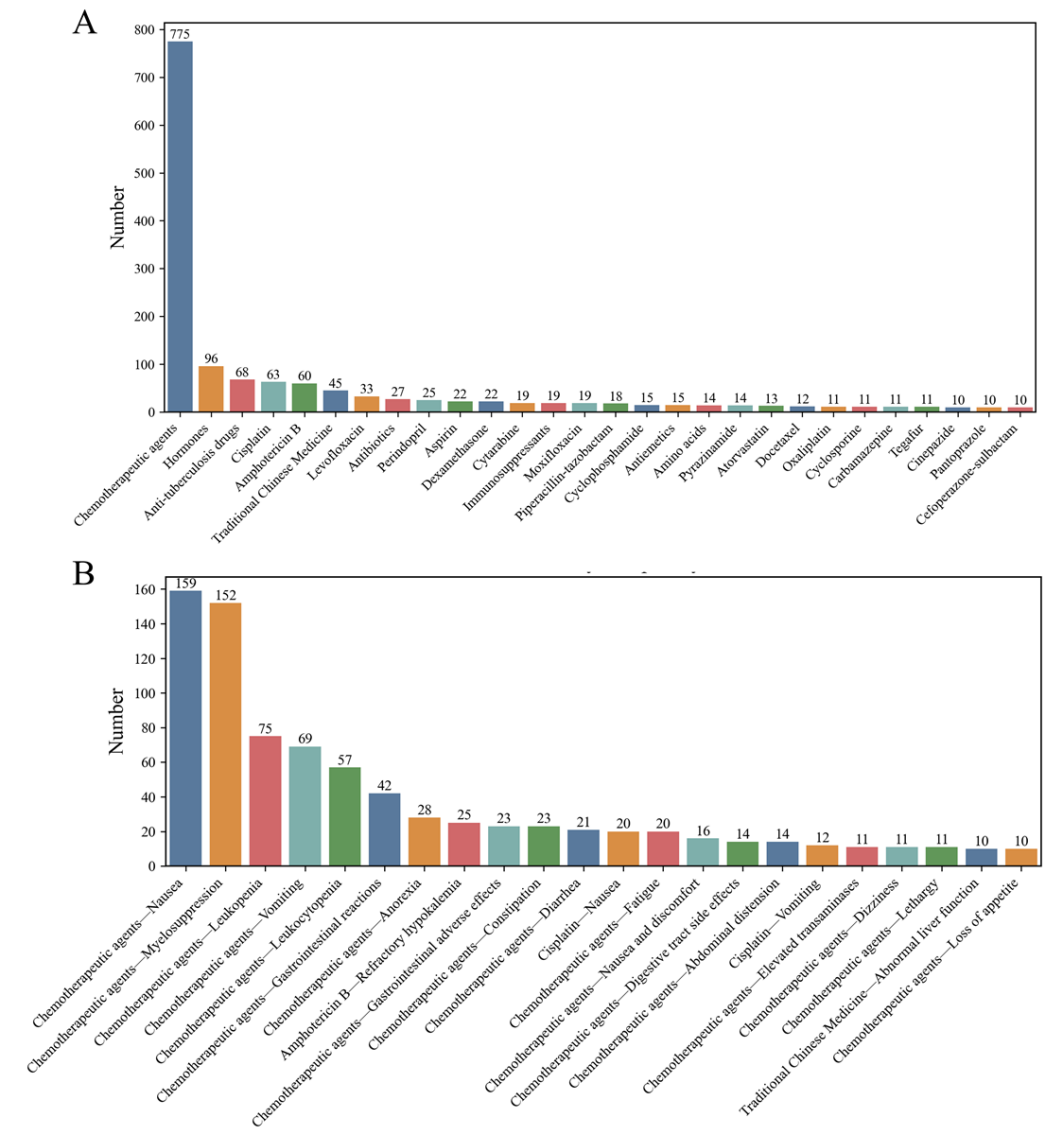
Frequency distribution of (A) drug entities and (B) adverse drug events in the initial positive reference set.

After a thorough manual review and annotation process, an initial analysis was conducted prior to data deduplication to understand the distribution characteristics of the data. A frequency analysis of drug entities and ADEs was conducted within the positive reference set. This dataset encompassed 748 unique drug entities, each of which appeared on average 3 times, with 28 entities exceeding 10 occurrences. Notably, chemotherapeutic agents represented the most common drug entity category, appearing 775 times (Figure 3A). This count included only instances in which the generic term itself was recorded in the annotation, whereas specific chemotherapy drugs were retained as distinct entities rather than aggregated under this term. Concurrently, 2668 unique ADE types were annotated, 22 of which appeared 10 or more times (Figure 3B). As detailed in the “Methods” section, to mitigate potential bias caused by this high frequency of redundant cases, both datasets underwent a rigorous deduplication and balancing process. This procedure ultimately yielded a refined standard reference dataset of 2510 records and an ADE knowledge base of 5144 records, which were subsequently used for all downstream model evaluations. A baseline RAG experiment using the full nondeduplicated knowledge base of 12,897 records showed slightly lower performance than the deduplicated knowledge base (Table S1 in Multimedia Appendix 1), supporting that limiting each drug entity to no more than 3 appearances improved retrieval diversity and reduced retrieval homogenization.

This work marks the first public release of a Chinese clinical note-based ADE research dataset, which is intended to be an open resource for the scientific community. The dataset is accessible at a GitHub repository [23]. Each record is characterized by 3 attributes: ID (a unique dataset identifier), Content (the clinical progress note text), and ADEs (annotations conforming to the ADE JSON schema specification).

### Overall Results

To ensure the reliability of the experimental results and the stability of the methodology, we used 1000 bootstrap resamples. In each round, 1000 samples were randomly selected with replacement from the standard reference dataset of 2510 samples, and the ADE identification method was evaluated on the resampled subset. The statistical results are summarized in Table 2 as bootstrap means with percentile-based 95% CI. Under the NAG prompt, all LLMs demonstrated strong ADE recognition performance, with both DeepSeek-V3 and ERNIE 3.5-8K achieving an overall *F*_1_-score of over 0.9. At the L2 and L3 matching levels, adoption of the SAG prompt improved the recognition performance of all models compared to the NAG prompt, indicating that optimizing prompt design and incorporating domain-specific knowledge generally enhances the model’s ability to recognize ADEs. Notably, applying the RAG prompt, which integrates retrieval information from a large-scale ADE case knowledge base, resulted in even greater performance improvements for all models compared to the SAG prompt. DeepSeek-V3 achieved the most outstanding recognition performance with an impressive overall *F*_1_-score of 0.9638 (95% CI 0.9541‐0.9727).

**Table 2.** Overall performance comparison of various LLMs^a^ on different matching levels and prompting strategies^b^.

Prompt, LLM, and level	Precision, mean (95% CI)	Recall, mean (95% CI)	*F*_1_-score, mean (95% CI)
NAG^c^			
ERNIE 3.5-8K			
L1	0.9494 (0.9317‐0.9678)	0.5951 (0.5620‐0.6260)	0.7315 (0.7054‐0.7554)
L2	0.9659 (0.9539‐0.9783)	0.8968 (0.8780‐0.9160)	0.9300 (0.9174‐0.9420)
L3	0.9669 (0.9553‐0.9789)	0.9247 (0.9080‐0.9420)	0.9453 (0.9345‐0.9560)
DeepSeek-V3			
L1	0.9288 (0.9061‐0.9534)	0.5143 (0.4800‐0.5480)	0.6619 (0.6305‐0.6928)
L2	0.9509 (0.9358‐0.9674)	0.7642 (0.7340‐0.7920)	0.8473 (0.8271‐0.8666)
L3	0.9559 (0.9425‐0.9707)	0.8544 (0.8300‐0.8780)	0.9022 (0.8870‐0.9179)
GPT-4o			
L1	0.9395 (0.9158‐0.9637)	0.3947 (0.3620‐0.4280)	0.5556 (0.5230‐0.5895)
L2	0.9575 (0.9402‐0.9738)	0.5723 (0.5400‐0.6060)	0.7162 (0.6888‐0.7432)
L3	0.9619 (0.9470‐0.9767)	0.6419 (0.6100‐0.6720)	0.7698 (0.7454‐0.7934)
SAG^d^			
ERNIE 3.5-8K			
L1	0.9178 (0.8944‐0.9403)	0.5674 (0.5340‐0.6020)	0.7011 (0.6733‐0.7282)
L2	0.9485 (0.9337‐0.9632)	0.9360 (0.9200‐0.9520)	0.9422 (0.9308‐0.9534)
L3	0.9499 (0.9358‐0.9641)	0.9644 (0.9520‐0.9780)	0.9571 (0.9475‐0.9663)
DeepSeek-V3			
L1	0.9724 (0.9576‐0.9863)	0.5425 (0.5100‐0.5760)	0.6963 (0.6684‐0.7234)
L2	0.9821 (0.9722‐0.9908)	0.8471 (0.8240‐0.8720)	0.9096 (0.8942‐0.9237)
L3	0.9833 (0.9741‐0.9914)	0.9080 (0.8900‐0.9260)	0.9441 (0.9333‐0.9556)
GPT-4o			
L1	0.9742 (0.9583‐0.9908)	0.4432 (0.4120‐0.4780)	0.6090 (0.5778‐0.6424)
L2	0.9821 (0.9709‐0.9937)	0.6440 (0.6120‐0.6760)	0.7778 (0.7534‐0.8019)
L3	0.9835 (0.9732‐0.9943)	0.6993 (0.6680‐0.7300)	0.8173 (0.7952‐0.8391)
RAG^e^			
ERNIE 3.5-8K			
L1	0.9439 (0.9251‐0.9623)	0.5878 (0.5540‐0.6220)	0.7243 (0.6986‐0.7500)
L2	0.9639 (0.9520‐0.9765)	0.9316 (0.9140‐0.9500)	0.9474 (0.9370‐0.9586)
L3	0.9648 (0.9530‐0.9773)	0.9569 (0.9420‐0.9700)	0.9608 (0.9519‐0.9700)
DeepSeek-V3			
L1	0.9686 (0.9525‐0.9851)	0.5482 (0.5160‐0.5820)	0.7000 (0.6727‐0.7270)
L2	0.9796 (0.9688‐0.9905)	0.8537 (0.8300‐0.8780)	0.9123 (0.8987‐0.9268)
L3	0.9816 (0.9716‐0.9916)	0.9466 (0.9320‐0.9620)	0.9638 (0.9541‐0.9727)
GPT-4o			
L1	0.9577 (0.9396‐0.9741)	0.5797 (0.5480‐0.6120)	0.7221 (0.6955‐0.7473)
L2	0.9710 (0.9586‐0.9819)	0.8565 (0.8340‐0.8800)	0.9101 (0.8958‐0.9241)
L3	0.9730 (0.9613‐0.9832)	0.9241 (0.9080‐0.9420)	0.9479 (0.9372‐0.9588)

a LLM: large language model.

b Values are presented as means with percentile-based 95% CIs from 1000 bootstrap resampling iterations. Prompting strategies evaluated include nonaugmented generation serving as a zero-shot baseline, static-augmented generation integrating static clinical diagnostic rules, and retrieval-augmented generation dynamically injecting the top 5 similar cases for context-aware, few-shot prompting. Matching levels are defined as L1 (exact match of sentences, drugs, and reactions), L2 (sentence-level match with drug or reaction discrepancies), and L3 (partial overlap of entities).

c NAG: nonaugmented generation.

d SAG: static-augmented generation.

e RAG: retrieval-augmented generation.

### Comparative Analysis of Prompts

**Figure 4.**
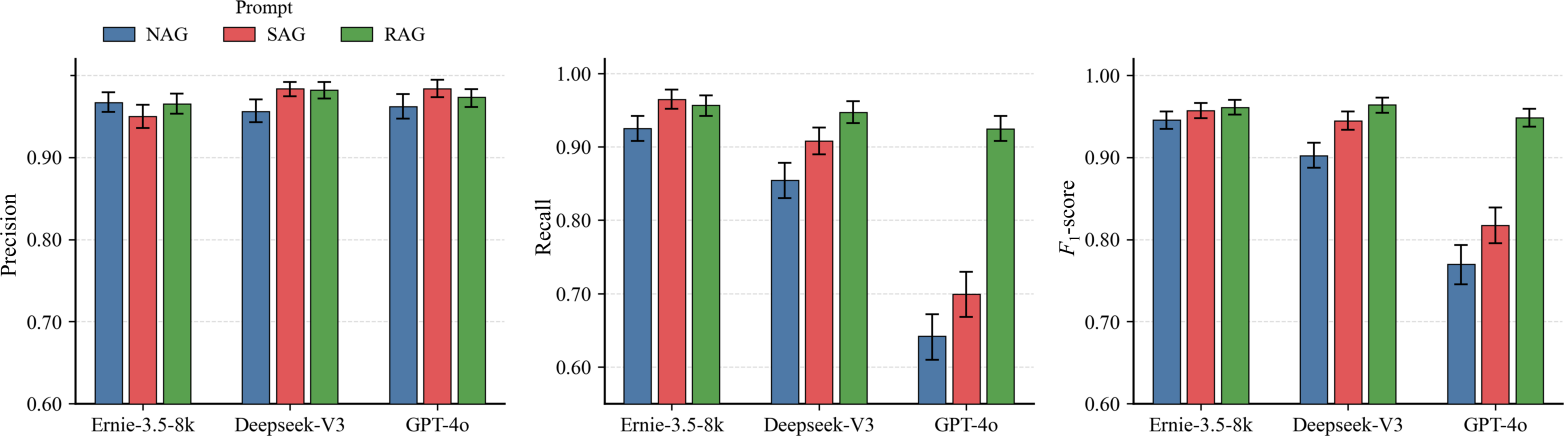
Performance of large language models in adverse drug event recognition across prompting strategies. Bar charts illustrate precision, recall, and *F*_1_-scores under nonaugmented generation, static-augmented generation, and retrieval-augmented generation prompts evaluated at the L3 matching level. Bars represent bootstrap mean values, and error bars indicate percentile-based 95% CIs. Knowledge augmentation significantly improves performance, with retrieval-augmented generation achieving average *F*_1_-scores >0.95 and substantially boosting GPT-4o’s recall. NAG: nonaugmented generation; RAG: retrieval-augmented generation; SAG: static-augmented generation.

Initially, an analysis of the ADE recognition instruction framework at the L3 level is presented. As illustrated in Figure 4, the comparison of recognition performance across various prompts at the L3 level is demonstrated for different LLMs. Compared with NAG, the SAG prompt increased the *F*_1_-score of DeepSeek-V3 from 0.9022 to 0.9441 (FDR *P*=.003) and GPT-4o from 0.7698 to 0.8173 (FDR *P*=.006). For ERNIE 3.5-8K, SAG produced only a modest increase from 0.9453 to 0.9571, which did not reach statistical significance in the paired bootstrap test (FDR *P*=.09). Building on the SAG prompt, the RAG prompt further augmented the comprehension capacity of the LLMs, yielding remarkable performance. DeepSeek-V3 and GPT-4o showed improvements of 1.97% (FDR *P*=.003) and 13.06% (FDR *P*=.003), respectively. These findings affirm the crucial role of augmenting domain knowledge in improving ADE recognition performance within mainstream LLMs and validate the effectiveness of the ADE case knowledge base designed in this study. The knowledge retrieval enhancement method was successful in significantly boosting ADE recognition performance. Ultimately, under the RAG prompt, mainstream LLMs exhibited remarkable improvement in ADE recognition, with average *F*_1_-scores exceeding 0.95.

Regarding precision (Figure 4), all models achieved a high level of precision under the NAG prompt, with the lowest precision reaching 95.59%. Minor fluctuations were subsequently observed under the SAG and RAG prompts, yet an overall upward trend in performance was evident. In terms of recall (Figure 4), ERNIE 3.5-8K and DeepSeek-V3 similarly achieved high recall rates under the NAG prompt, reaching 92.47% and 85.44%, respectively. In contrast, GPT-4o exhibited a significantly lower recall rate of only 64.19%, indicating a substantial proportion of ADEs went unrecognized. However, under the SAG prompt, GPT-4o’s recall rate increased to 69.93%, and under the RAG prompt, it surged to 92.41%, representing an improvement of almost 30% (FDR *P*=.003). This corroborates the effectiveness of the knowledge retrieval enhancement method used in this experiment. It also highlights that while GPT-4o has considerable generative capabilities, its lack of domain-specific understanding limits its performance. However, upon incorporating contextual knowledge, its performance was substantially enhanced, ultimately bringing its overall *F*_1_-score in line with those of ERNIE 3.5-8K and DeepSeek-V3.

### Comparative Analysis of LLMs

**Figure 5.**
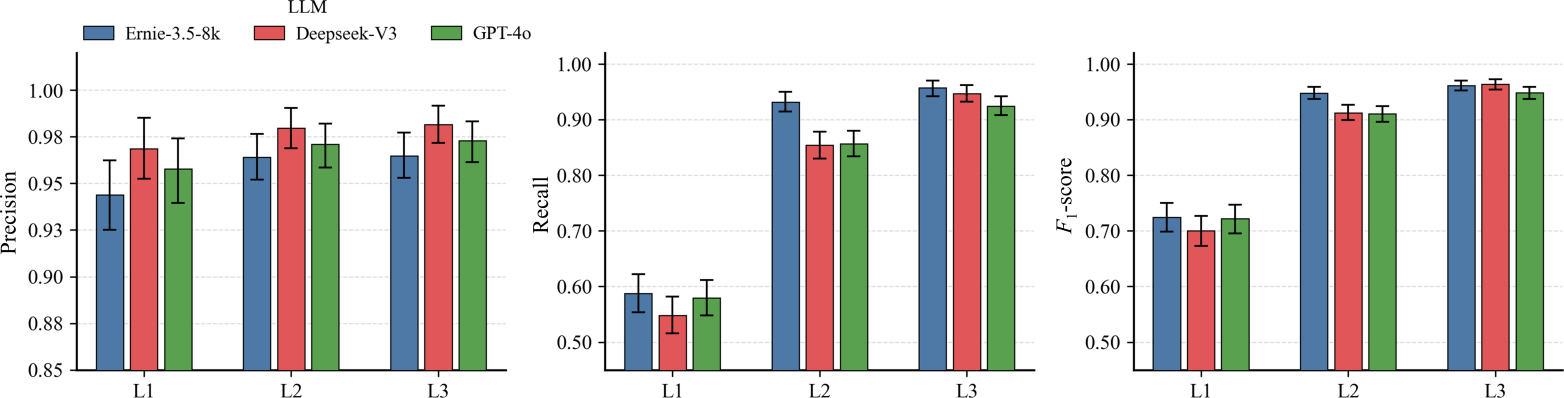
Performance comparison of various large language models. Bar charts compare precision, recall, and *F*_1_-scores for 3 large language models using the retrieval-augmented generation prompt at L1, L2, and L3 levels. Bars represent bootstrap mean values, and error bars indicate percentile-based 95% CIs. For *F*_1_-scores, ERNIE 3.5-8K marginally led at L1 (72.43%) and L2 (94.74%), whereas DeepSeek-V3 led at L3 (96.38%). LLM: large language model.

We further analyzed the variations in ADE recognition performance across different models. Taking the RAG prompt as an example, the comparative analysis at different matching levels is illustrated in Figure 5. With regard to precision, all 3 models demonstrated consistently stable performance across the L1, L2, and L3 levels, with each achieving a precision rate of over 94%. In terms of recall, ERNIE 3.5-8K slightly outperformed the other 2 models. Regarding the *F*_1_-score, there was minimal variation in the models’ recognition performance: at the L1 level, ERNIE 3.5-8K marginally surpassed the others with an *F*_1_-score of 72.43%, though this advantage was not statistically significant compared to GPT-4o (FDR *P*=.86); at the L2 level, ERNIE 3.5-8K led with a score of 94.74%, significantly outperforming both DeepSeek-V3 and GPT-4o (both FDR *P*=.003). At the L3 level, DeepSeek-V3 slightly outperformed the others with an *F*_1_-score of 96.38% (FDR *P*=.01 compared to GPT-4o, but no significant difference compared to ERNIE 3.5-8K, FDR *P*=.68). Thus, under the RAG prompt, the overall performance of LLMs in ADE recognition for Chinese clinical progress notes is relatively consistent, with each model displaying robust semantic comprehension and generation capabilities.

### Comparative Analysis of Various Matching Levels

**Figure 6.**
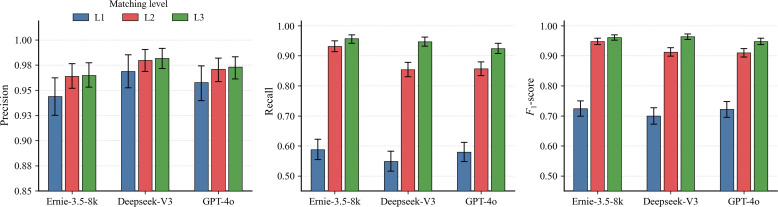
Performance comparison of large language models on different matching levels. Performance of large language models across L1, L2, and L3 matching levels. Evaluated using the retrieval-augmented generation prompt, bar charts demonstrate a significant upward trend in precision, recall, and *F*_1_-scores from L1 to L3. Bars represent bootstrap mean values, and error bars indicate percentile-based 95% CIs. Substantial recall improvements from L1 to L2 notably drive overall *F*_1_-score increases.

Taking the RAG prompt analysis as an example, we examined the models’ performance disparities across different matching levels (Figure 6). The performance of each model showed an upward trend from L1 to L2, and subsequently to L3 (all FDR *P*<.05), with the most significant improvements in recall value. For instance, at L2, ERNIE 3.5-8K demonstrated a 34.38% improvement over L1, reaching 93.16%. DeepSeek-V3 showed a 30.55% increase, achieving 85.37%; and GPT-4o exhibited a 27.68% enhancement, attaining 85.65%. These results emphasize the important role of accurate entity recognition in ADE detection. Regarding the *F*_1_-score, the substantial increase in recall at L2 led to a similar upward trend across all matching levels.

Given that the most substantial performance variance occurred at the L2 level, we undertook a granular analysis of these cases. Focusing on the DeepSeek-V3 model as a representative example, we identified 376 instances of L2-level matches within a standard reference corpus of 2510 entries. Our analysis of these instances revealed 2 main error categories: anomalous recognition of drug entities and anomalous recognition of adverse reaction entities (Table 3). The former category was further stratified into four subtypes: (1) omission of the correct drug entity, (2) commission of an incorrect drug entity, (3) misidentification of the drug entity, and (4) lack of precision in boundary detection. Similarly, anomalies in adverse reaction entity recognition comprised: (1) entity omission, (2) entity commission, (3) failure to disaggregate compound entities, and (4) imprecise boundary detection. Quantitative analysis of these error subtypes highlighted the omission of adverse reaction entities as the predominant failure mode, accounting for around 40.7% of the L2-level discrepancies.

**Table 3.** Case analysis of anomalies in entity recognition.

Categories and specific reasons	Examples of cases
Abnormal drug entities	
Missed entities	#13806**:** Failure to identify ARB^a^ drug entities
Incorrect additional entities	#15018**:** Among the 2 identified drug entities, “amino acids” and “compound amino acids,” only “compound amino acids” is required. #12731**:** An incorrect drug entity “statin drugs” has been identified.
Misidentified	#7435**:** Two incorrect drug entities have been identified: “reduced glutathione” and “magnesium isoglycyrrhizinate.” #13961**:** Two incorrect drug entities have been identified: “methylprednisolone powder for injection” and “cyclosporine.”
Inaccurate entities	#4316**:** The identified drug entity is “chemotherapy drugs,” but it needs to be specified that the chemotherapy drug is “ifosfamide.” #4176**:** The identified drug entity is “hemostatic drugs,” but it needs to be specified that the actual drug entity is “pituitrin.” #7293**:** The identified drug entity is “chemotherapy drugs,” but it needs to be specified that the actual drug entities are “oxaliplatin” and “fluorouracil.”
Abnormal reaction entities	
Missed entities	#12191**:** The adverse reaction entity “elevated liver transaminases” was not identified. #6967**:** The adverse reaction entity “elevated alanine aminotransferase” was not identified. #4782**:** The adverse reaction entity “low platelet count” was not identified. #13993**:** The adverse reaction entity “myalgia” was not identified. #14145**:** The adverse reaction entity “rash” was not identified. #3341**:** The adverse reaction entity “iodine allergy” was not identified.
Incorrect additional entities	#11640**:** An unconfirmed adverse reaction entity such as “vomiting” has been identified. #14307**:** An unconfirmed adverse reaction entity, “liver dysfunction,” has been identified. #3099**:** An unknown adverse reaction entity, “allergic reaction,” has been identified.
Unsplited entities	#4444**:** The entity “palpitations and shortness of breath” has been identified and needs to be split into 2 separate entities: “palpitations” and “shortness of breath.” #14307**:** The entity “nausea and vomiting” has been identified and requires splitting into 2 separate entities: “nausea” and “vomiting.” #4698**:** The entity “chills and fever” has been identified and needs to be divided into two separate entities: “chills” and “fever.”
Inaccurate entities	#4036**:** The entity “abnormal liver function” has been identified, but it needs to be clarified that the specific manifestations of this abnormal liver function are “elevated alanine aminotransferase” and “elevated aspartate aminotransferase.” #3073**:** The adverse reaction entity description “glucose+4≥55 mmol/L” has been identified, but it needs to be converted into a clearly defined entity: “elevated urinary glucose.”

a ARB: angiotensin II receptor blocker.

### Error Cases Analysis and Summary

**Figure 7.**
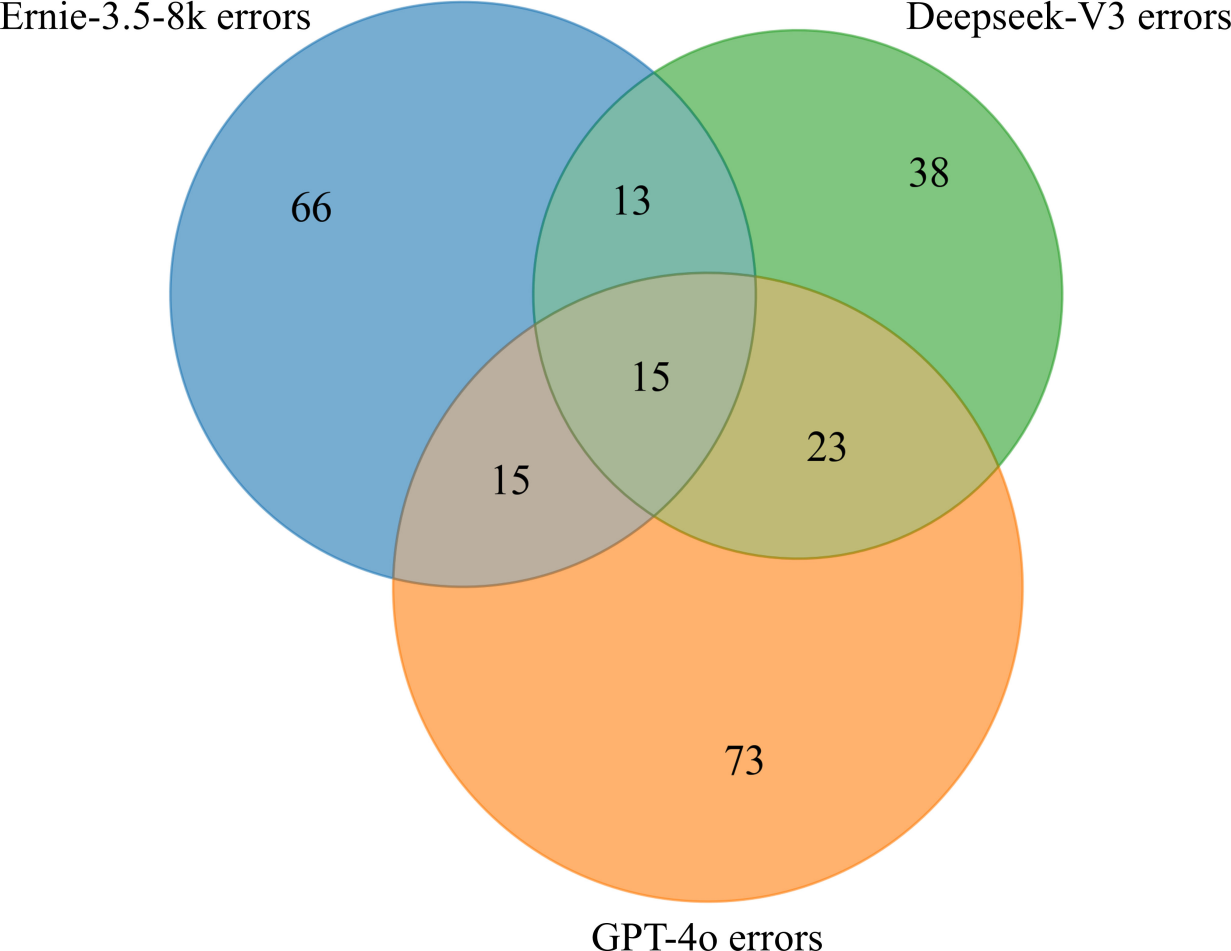
Venn diagram of overlapping recognition errors across 3 large language models.

A statistical analysis of error instances revealed that ERNIE 3.5-8K incorrectly handled 66 case records alone, DeepSeek-V3 incorrectly handled 38 case records alone, and GPT-4o incorrectly handled 73 case records alone (Figure 7). Notably, 15 critical records posed recognition challenges for all 3 models. This suggests that, despite the integration of 3 LLMs in a composite approach, these ADE remain unrecognized. A detailed etiological analysis of these 15 universally misidentified cases partitioned the errors into 2 principal categories: failures of omission (failure to identify a true ADE) and failures of commission (identification of an erroneous ADE; Table 4). The latter constituted the majority of errors, accounting for approximately 80% of instances. The primary drivers of these commission errors were identified as (1) non–drug-related adverse events; (2) the incorrect classification of prophylactic, precautionary, or advisory medication mentions; and (3) the misinterpretation of historical ADE descriptions as contemporary events. Further investigation demonstrated that these erroneous identifications could be rectified by augmenting the models with a domain-specific ADE knowledge base via a RAG framework. This underscores the profound efficacy of incorporating a curated knowledge repository and RAG methods to enhance the precision of ADE detection.

**Table 4.** Analysis of misidentifications by 3 LLMs^a^.

Number	Case ID	Reasons for the errors
1	#3160	Unrecognized ADE^b^: “Metabolic alkalosis with compensatory response, suspected diuretic-related.”
2	#12251	Unrecognized ADE: “The suspected cause of altered mental status includes intracranial edema or drug-induced toxicity combined with uremic toxin damage.”
3	#3310	Unrecognized ADE: “Cardiac troponin I negative, electrolytes normal; drug-related etiology suspected.”
4	#4650	Misidentified ADE: “The instructor noted that sodium nitroprusside had been administered for 10 days and discontinued to prevent long-term adverse effects, with isosorbide dinitrate substituted for vasodilation, antihypertension, and heart failure control.” It pertains to prophylactic drug discontinuation due to anticipated adverse reactions, rather than an actual ADE.
5	#3896	Misidentified ADE: “The patient received 300 mL of O(+) frozen plasma yesterday and reported generalized pruritus, pain, and numbness post-transfusion.” It pertains to a transfusion-related reaction, not a drug-induced adverse event.
6	#11788	Misidentified ADE: “E4A+CO2P: K+ 5.74 mmol/L indicates hyperkalemia, suspected to result from recent repeated blood transfusions.” It pertains to a transfusion-associated electrolyte disturbance, not a drug-induced adverse event.
7	#4651	Misidentified ADE: “Yesterday, during the infusion of 600 mL of O-Rh(D)-positive frozen plasma of the same blood type, the patient developed pruritus.” It pertains to a transfusion-associated allergic reaction, not a drug-induced adverse event.
8	#12270	Misidentified ADE: “Liver function tests revealed elevated transaminases; Shu Ganning Injection was administered for hepatic protection.” It pertains to medication management rather than an ADE.
9	#4527	Misidentified ADE: “Considering the neurotoxic potential of vincristine causing peripheral neuropathy (numbness in hands and feet), vitamin B1 tablets 20mg orally were added prophylactically today.” It constitutes a description of prophylactic medication for adverse reaction prevention, rather than an actual ADE.
10	#11746	Misidentified ADE: “Given the patient’s clinical deterioration with potential pulmonary hemorrhage relapse,(LMWH)^c^ was discontinued, and airway status monitoring was initiated.” It constitutes proactive ADE prevention measures rather than an actual ADE.
11	#7332	Misidentified ADE: “Patient’s family was informed of significant antifungal drug-related risks, including hepatorenal toxicity, leukopenia or thrombocytopenia, and adverse reactions such as chills, high fever, and thrombophlebitis.” It constitutes pharmacovigilance counseling rather than an actual ADE.
12	#2939	Misidentified ADE: “Patient demonstrated clinical improvement post-intravenous doxofylline and ambroxol administration (paroxysmal wheezing alleviated), with residual symptoms of mild white sputum production and pruritic trunk rash.” It pertains to therapeutic response assessment rather than an ADE.
13	#5973	Misidentified ADE: “Amikacin administered for 7 days; discontinued today to prevent potential renal impairment.” It constitutes a prophylactic toxicity narrative rather than an ADE.
14	#11731	Misidentified ADE: “Patient exhibits hypoglycemia during early morning and nighttime; insulin dose reduced to 10 units subcutaneous injection at bedtime today.” The full clinical context did not provide sufficient causal evidence linking the hypoglycemia episode to insulin as a dose-dependent ADE.
15	#3074	Misidentified ADE: “Patient on self-administered (ART)^d^ with history of (DILI)^e^.” It represents a preexisting adverse drug reaction.

a LLM: large language model.

b ADE: adverse drug event.

c LMWH: low-molecular-weight heparin.

d ART: antiretroviral therapy.

e DILI: drug-induced liver injury.

### Subanalysis of Complex ADE Linguistic Contexts

To empirically evaluate the model’s ability to handle complex clinical language, we constructed a targeted subset of 100 clinical notes containing 3 challenging ADE-related contexts: negation, abbreviations, and temporal and causal descriptions (Table S2 in Multimedia Appendix 1). Using DeepSeek-V3 under the RAG prompting strategy, the model achieved a precision of 0.9167, recall of 0.9429, and *F*_1_-score of 0.9296 on this subset. These findings indicate that the RAG framework can effectively process negated ADE descriptions, abbreviated drug expressions, and historical or risk-monitoring contexts, supporting its robustness in complex clinical narratives.

### Performance Evaluation on Real-World Dataset

To validate clinical utility under real-world conditions, DeepSeek-V3 was evaluated using 1000 random resampling iterations from 19,983 uncurated clinical progress notes, with 1000 notes sampled in each iteration (Table 5). At the L3 level, RAG achieved an *F*_1_-score of 0.8159 (95% CI 0.7736‐0.8598), compared with 0.6380 (95% CI 0.5759‐0.6942) for NAG and 0.8134 (95% CI 0.7600‐0.8627) for SAG. Despite relatively low precision under this low-prevalence setting, RAG demonstrated strong clinical utility, achieving a recall of 0.9448, *F*_2_-score of 0.8885, G-mean of 0.9631, Cohen κ of 0.8057, and specificity of 0.9821. These findings confirm that while absolute *F*_1_-scores slightly adjust due to the natural data imbalance, integrating a curated knowledge base via RAG remains a highly effective and practically viable approach for clinical ADE identification.

**Table 5.** Performance of DeepSeek-V3 on real-world uncurated clinical progress notes^a^.

Prompt and level	Precision, mean (95% CI)	Recall, mean (95% CI)	*F*_1_-score, mean (95% CI)	Specificity, mean (95% CI)	*F*_2_-score, mean (95% CI)	G-mean, mean (95% CI)	Cohen κ, mean (95% CI)
NAG^b^							
L1	0.3836 (0.3051‐0.4603)	0.5151 (0.3696‐0.6522)	0.4391 (0.3400‐0.5345)	0.9602 (0.9539‐0.9665)	0.4816 (0.3571‐0.6009)	0.7015 (0.5960‐0.7931)	0.4080 (0.3055‐0.5079)
L2	0.4810 (0.4225‐0.5375)	0.7640 (0.6522‐0.8696)	0.5899 (0.5167‐0.6557)	0.9602 (0.9539‐0.9665)	0.6831 (0.5882‐0.7693)	0.8559 (0.7900‐0.9152)	0.5653 (0.4876‐0.6349)
L3	0.5093 (0.4594‐0.5617)	0.8549 (0.7603‐0.9348)	0.6380 (0.5759‐0.6942)	0.9602 (0.9539‐0.9665)	0.7524 (0.6679‐0.8206)	0.9056 (0.8517‐0.9489)	0.6158 (0.5498‐0.6757)
SAG^c^							
L1	0.6265 (0.5385‐0.7084)	0.5423 (0.4130‐0.6957)	0.5801 (0.4691‐0.6882)	0.9845 (0.9811‐0.9885)	0.5565 (0.4338‐0.6858)	0.7291 (0.6373‐0.8262)	0.5615 (0.4472‐0.6730)
L2	0.7247 (0.6723‐0.7819)	0.8455 (0.7391‐0.9348)	0.7799 (0.7158‐0.8381)	0.9845 (0.9811‐0.9885)	0.8178 (0.7296‐0.8943)	0.9120 (0.8529‐0.9607)	0.7684 (0.7016‐0.8293)
L3	0.7385 (0.6897‐0.7925)	0.9062 (0.8261‐0.9783)	0.8134 (0.7600‐0.8627)	0.9845 (0.9811‐0.9885)	0.8665 (0.7983‐0.9259)	0.9443 (0.9012‐0.9818)	0.8034 (0.7475‐0.8554)
RAG^d^							
L1	0.5947 (0.5128‐0.6758)	0.5471 (0.4130‐0.6957)	0.5687 (0.4578‐0.6739)	0.9821 (0.9780‐0.9864)	0.5553 (0.4318‐0.6809)	0.7315 (0.6369‐0.8257)	0.5490 (0.4346‐0.6582)
L2	0.6970 (0.6481‐0.7586)	0.8524 (0.7391‐0.9353)	0.7663 (0.7000‐0.8257)	0.9821 (0.9780‐0.9864)	0.8156 (0.7295‐0.8921)	0.9146 (0.8529‐0.9609)	0.7539 (0.6843‐0.8159)
L3	0.7185 (0.6724‐0.7759)	0.9448 (0.8696‐1.0000)	0.8159 (0.7736‐0.8598)	0.9821 (0.9780‐0.9864)	0.8885 (0.8333‐0.9350)	0.9631 (0.9251‐0.9916)	0.8057 (0.7611‐0.8521)

a A total of 1000 random resampling iterations were conducted from 19,983 raw clinical progress notes. The average adverse drug event prevalence was approximately 4.6%. Values are presented as means with percentile-based 95% CIs across 1000 resampling iterations. Prompting strategies evaluated include nonaugmented generation serving as a zero-shot baseline, static-augmented generation integrating static clinical diagnostic rules, and retrieval-augmented generation dynamically injecting top-5 similar cases for context-aware, few-shot prompting. Matching levels are defined as L1 (exact match of sentences, drugs, and reactions), L2 (sentence-level match with drug or reaction discrepancies), and L3 (partial overlap of entities).

b NAG: nonaugmented generation.

c SAG: static-augmented generation.

d RAG: retrieval-augmented generation.

## Discussion

### Principal Findings

This study investigates the identification of ADEs from clinical course records by synergistically integrating LLMs with RAG technology. Using a progressive research methodology, we demonstrate that the incorporation of a RAG framework substantially enhances the performance of LLMs in the ADE recognition task. Comparative experimental analysis reveals that our proposed RAG solution, which leverages a curated ADE knowledge repository, achieves superior performance across precision, recall, and *F*_1_-score metrics, thereby validating its considerable utility in the domain of medical text analysis. Furthermore, this work contributes a novel benchmark corpus, addressing the existing scarcity of annotated data for ADE extraction in the Chinese language.

In the absence of knowledge augmentation (using NAG prompt), the various LLMs exhibited significant performance disparities in ADE recognition. This variability likely stems from differences in their foundational training corpora, including the breadth of medical data coverage, divergent parameter optimization strategies, and the nuanced depth of their comprehension of Chinese clinical terminology [24]. Among the models evaluated, DeepSeek-V3 demonstrated optimal recognition performance under the RAG prompt. DeepSeek-V3, which claimed performance competitive with GPT-4o shortly after its release in January 2025, has rapidly gained global prominence [25]. Unlike the proprietary GPT-4o, DeepSeek-V3 is an open-source model that permits local deployment. In a local deployment, its parameter weights can remain insulated from alterations in cloud infrastructure or API updates, thereby supporting long-term consistency and reproducibility while protecting patient data privacy. These advantages create a favorable pathway for the future deployment and external validation of this architectural framework within local health care institutions. However, this study used the cloud API version of DeepSeek-V3 to improve experimental efficiency and standardize batch inference. Local deployment therefore remains a future pathway for privacy-preserving implementation, rather than the deployment mode used in this study.

In the real-world low-prevalence evaluation, the L3 *F*_1_-score differed only marginally between SAG and RAG (0.8134 vs 0.8159), likely because the 2 evaluation settings differed substantially in data distribution. In the same uncurated setting, SAG slightly exceeded RAG in *F*_1_-score at L1 (0.5801 vs 0.5687) and L2 (0.7799 vs 0.7663). The curated reference set was relatively balanced and enriched for ADE-positive cases, allowing dynamic retrieval to provide closely matched examples and entity-level contextual cues that improved drug and reaction recognition. By contrast, routine clinical notes had a low ADE prevalence and were dominated by negative records. In this setting, the static clinical rules embedded in SAG already captured common negative contexts, including historical ADE descriptions, prophylactic medication use, and routine monitoring statements. This strong baseline specificity limited the incremental *F*_1_-score gain from dynamic retrieval. However, RAG improved recall at the L3 level from 0.9062 to 0.9448, indicating better detection of rare positive ADE cases. Thus, in low-prevalence clinical data, RAG primarily enhances positive-case recall, underscoring its capacity to effectively mitigate the risk of underreporting ADEs.

Our empirical findings demonstrate that the performance of a conventional, pure language model is markedly inferior to that of a variant augmented with a RAG framework. This outcome aligns with the established theoretical premise that the integration of domain-specific knowledge substantially enhances the capabilities of large models. In contrast to fine-tuning methodologies, RAG obviates the need for voluminous training corpora and protracted training cycles, and circumvents the laborious process of creating contemporary annotated datasets or engaging in repetitive model retraining to assimilate updated, customized knowledge [26]. The paramount advantage of the RAG model resides in its profound adaptability [19], enabling the continuous assimilation of the latest information on ADE cases—a feature of particular salience within the dynamic landscape of medicine and pharmacology, where novel therapeutics are developed at a rapid pace. Consequently, when information regarding an ADE for a newly marketed drug becomes available, it can be seamlessly incorporated into the ADE knowledge repository, facilitating dynamic updates without necessitating extensive modifications to the core model. Furthermore, RAG’s capacity to reference and cite antecedent ADE cases enhances the verifiability and perceived relevance of its outputs, thereby effectively mitigating the phenomenon of “confabulation,” or artifactual generation, which frequently plagues the application of LLMs in medical contexts, while concurrently improving model transparency [27]. An error analysis revealed 15 common errors that were uniformly misidentified by all tested models, exposing the inherent limitations of LLMs in processing specific complexities within clinical texts. Critically, we demonstrated that these identification failures could be rectified by supplementing the model’s context with pertinent ADE case knowledge via the RAG architecture. This not only furnishes a definitive pathway for augmenting the precision of LLMs in specialized domains but also underscores the intrinsic value of the ADE knowledge repository. Future work should therefore be directed towards the construction of a more comprehensive repository that encompasses not only ADE case reports but also multifaceted information from product information, clinical practice guidelines, and disease manifestations.

### Comparison With Previous Studies

To date, systematic investigations into the application of LLMs for general ADE information recognition remain nascent, with most research focusing on narrowly defined ADEs. For instance, Cheligeer et al [28] evaluated 4 open-source LLMs in the detection of pulmonary embolism from narrative electronic medical records, while Li et al [29] demonstrated the efficacy and robustness of LLMs in extracting postvaccination adverse events from diverse data sources, including the VAERS (US Centers for Disease Control and Prevention and US Food and Drug Administration), Twitter (Twitter, Inc), and Reddit (Reddit, Inc). Our research, in contrast, underscores the broader potential of LLMs to identify all types of ADEs within unstructured Chinese clinical texts. The best-performing RAG-enhanced LLM achieved an *F*_1_-score of 96.38%, which is higher than the performance reported in some previous deep learning–based ADE extraction studies [30]. However, because these studies used different datasets, annotation criteria, and evaluation settings, this comparison should be interpreted only as indirect evidence rather than a head-to-head demonstration of superiority. Nevertheless, our findings suggest that RAG-enhanced LLMs represent a promising framework for ADE extraction, with potential advantages in contextual reasoning, prompt-based adaptation, and flexible knowledge updating [31]. Moreover, LLMs obviate the need for extensive labeled datasets and are amenable to continuous refinement as new data becomes available [32].

### Clinical Application

The methodologies and results presented herein possess considerable potential for translation into clinical practice, where they could elevate the standards of ADE surveillance and reporting and advance pharmacovigilance and drug safety research. Moreover, our approach can be embedded within Clinical Decision Support Systems. Such integration would synergistically combine ADE information with other relevant patient data streams (such as laboratory results, imaging data, and demographic information) to provide clinicians with a more holistic and precise patient assessment, thereby guiding more timely and judicious clinical decision-making. To further enhance the practical utility of our model, we are currently developing a web-based platform, which will feature a user-friendly interface allowing clinicians to query for ADE information within texts through simple conversational input.

### Limitations and Future Work

This study has several limitations. First, the knowledge repository was constructed from a single data source, which may result in an incomplete representation of all potential ADEs. Crucially, evaluating models on the same data distribution as the knowledge base risks overfitting and implicit data leakage within the RAG framework, limiting external validity. Multi-institutional validation, using independent data and varied clinical note formats, is therefore essential to verify real-world generalizability. In the future, the integration of multisource information, including pharmaceutical package inserts and expert-derived knowledge, would likely yield a substantial improvement in model performance.

Second, given the rapid pace of technological iteration in LLMs, the models used in this research may not represent the latest state-of-the-art (eg, the most recent Gemini [Google LLC] or Claude architectures [Anthropic PBC]). While larger-parameter models may offer performance gains, such enhancements must be carefully weighed against the concomitant increase in computational resource expenditure and memory requirements, which presents a significant challenge in resource-constrained settings.

Third, although the RAG-enhanced LLMs achieved promising performance in ADE identification, this study did not include a direct head-to-head comparison with standard clinical natural language processing baselines, such as fine-tuned clinical Bidirectional Encoder Representations from Transformers (BERT)–based models. Therefore, our findings should be interpreted as evidence of the feasibility and potential utility of the proposed framework, rather than definitive evidence of superiority over traditional deep learning approaches. Future work will include direct comparisons with conventional clinical natural language processing models on the same curated dataset and further explore LLM fine-tuning strategies to rigorously evaluate and optimize ADE identification performance.

Fourth, all clinical notes were strictly deidentified, preventing the extraction and reporting of baseline patient demographics. Despite this limitation, the generalizability of our findings is preserved through the random sampling of heterogeneous data.

Fifth, although the RAG model achieved high specificity (0.9821) in the real-world evaluation, this corresponds to an approximate false-positive rate of 1.79%. At the observed ADE prevalence of about 4.6%, this would translate into roughly 17 falsely flagged ADE-negative notes per 1000 screened notes, which could increase manual review burden or alert fatigue if deployed without clinician oversight. Future work should calibrate decision thresholds, retrieval filtering, human-in-the-loop review, and iterative knowledge-base expansion to balance sensitivity against false-alert burden while further improving RAG effectiveness and reducing hallucination-related false positives.

Sixth, all performance metrics in this study were computed at the document level. This evaluation strategy is clinically meaningful for screening whether a note contains ADE information, but document-level precision does not capture entity-level overextraction within otherwise ADE-positive notes. Future work should therefore supplement document-level evaluation to more comprehensively assess extraction granularity and hallucinated entities within positive notes.

In light of these constraints, our future research will explore the incorporation of knowledge graph embedding techniques [33]. By leveraging graph-based structural reasoning, this approach could facilitate the logical inference of ADE knowledge not explicitly contained within the existing database, thereby empowering users to identify potential ADEs with greater accuracy. To further enhance the clinical utility of the proposed system, future investigations should focus on expanding the ADE knowledge base framework and evaluating the system’s performance in diverse, real-world clinical settings. To facilitate this translation, we recently developed ADESys [34]. Future work will leverage this system for multicenter external validation to enhance our method’s robustness and translational value. Such efforts are essential to reducing ADE underreporting and increasing the overall efficiency of pharmacovigilance research.

### Conclusion

This study evaluated the effectiveness of a knowledge base-driven RAG framework in identifying ADE information from Chinese clinical narratives. The experimental results across 3 LLMs (DeepSeek-V3, ERNIE 3.5-8K, and GPT-4o) indicate that the RAG strategy consistently improves identification performance compared to NAG and SAG approaches. Furthermore, this research establishes a valuable benchmark, addressing the critical gap in ADE corpora within the Chinese language domain.

## Supplementary material

10.2196/84086 Multimedia Appendix 1 Supplementary figures, including baseline retrieval-augmented generation performance using the full knowledge base, an empirical subanalysis of complex adverse drug event linguistic contexts, the standardized adverse drug event JSON schema and annotation example, the prompt engineering framework, and illustrative examples of L1-L3 recognition levels.

## Abbreviations

ADE: adverse drug event
BERT: Bidirectional Encoder Representations from Transformers
FDR: false discovery rate
LLM: large language model
NAG: nonaugmented generation
RAG: retrieval-augmented generation
SAG: static-augmented generation
