# Supplementary material for: Intelligent Framework for Adverse Drug Event Identification Using Large Language Models and Retrieval-Augmented Generation: Development and Evaluation Study
Source: J Med Internet Res. 2026 Aug 10;28:e84086. doi: 10.2196/84086 (PMC13456140; doi:10.2196/84086)
Supplement: Supplementary file 1 [file jmir-v28-e84086-s001.docx]

**Table S1**. Baseline performance of RAG using the full non-deduplicated 12,897-record knowledge base. Values are presented as means with percentile-based 95% confidence intervals from 1,000 bootstrap resampling iterations. Matching levels are defined as L1, L2, and L3. LLM: large language model; RAG: retrieval-augmented generation.

| LLM | Level | Precision | Recall | F1 |
| --- | --- | --- | --- | --- |
| Ernie-3.5-8k | L1 | 0.8819 (0.8555–0.9077) | 0.5681 (0.5275–0.6075) | 0.6908 (0.6584–0.7232) |
|  | L2 | 0.9249 (0.9073–0.9416) | 0.9359 (0.9150–0.9550) | 0.9303 (0.9168–0.9434) |
|  | L3 | 0.9269 (0.9100–0.9432) | 0.9644 (0.9500–0.9800) | 0.9452 (0.9338–0.9571) |
| Deepseek-V3 | L1 | 0.9531 (0.9339–0.9726) | 0.5479 (0.5075–0.5900) | 0.6956 (0.6591–0.7298) |
|  | L2 | 0.9694 (0.9573–0.9827) | 0.8535 (0.8225–0.8850) | 0.9077 (0.8889–0.9263) |
|  | L3 | 0.9721 (0.9608–0.9840) | 0.9465 (0.9250–0.9675) | 0.9591 (0.9476–0.9709) |
| GPT-4o | L1 | 0.9412 (0.9176–0.9639) | 0.5781 (0.5375–0.6200) | 0.7162 (0.6846–0.7470) |
|  | L2 | 0.9597 (0.9444–0.9761) | 0.8554 (0.8250–0.8875) | 0.9038 (0.8865–0.9215) |
|  | L3 | 0.9625 (0.9478–0.9782) | 0.9220 (0.8975–0.9475) | 0.9418 (0.9286–0.9548) |

**Table S2**. Empirical sub-analysis of complex ADE linguistic contexts using Deepseek-V3 with RAG. A targeted subset of 100 clinical notes was constructed to evaluate whether the model could handle challenging ADE-related linguistic phenomena, including negation, abbreviations, and temporal/causal contexts. Representative phrases are translated from the original Chinese clinical notes. ADE: adverse drug event; RAG: retrieval-augmented generation.

| Complex ADE context | No. of notes | Representative examples | Expected interpretation |
| --- | --- | --- | --- |
| Negation | 40 | a) “The chemotherapy was completed smoothly, and no obvious adverse reaction was observed.” b) “After BB chemotherapy, there was no vomiting, diarrhea, chills, convulsions, dyspnea, or other adverse reactions.” c) “PD-1 was infused smoothly, with no chills, fever, or other adverse reactions observed.” | ADE-related symptoms were explicitly negated, these notes should be classified as ADE-negative. |
| Abbreviations | 35 | a) “WBC low was considered chemotherapy-induced myelosuppression.” b) “After PD-1 treatment, the patient developed rash, difficulty opening the eyes, dysphagia, and mild dyspnea.” c) “Leukopenia was considered to be caused by intraoperative MTX use.” | The model should correctly interpret both abbreviated drug/regimen expressions and abbreviated ADE manifestations, including WBC, PD-1, and MTX abnormalities. |
| Temporal/causal context | 25 | a) “The patient had a previous history of penicillin and streptomycin allergy.” b) “The patient had a history of drug-induced liver injury before admission.” c) “Regular liver function, thyroid function, and chest CT monitoring were recommended to evaluate possible amiodarone adverse effects.” | Historical ADEs, risk warnings, or monitoring recommendations should not be misclassified as current ADE events unless an active ADE is described. |
| Overall performance | 100 | Deepseek-V3 with RAG | Precision = **0.9167**; Recall = **0.9429**; F1 score = **0.9296** |

**Figure S1.** JSON schema of ADE. This schema defines a standardized array format requiring three mandatory fields: the original clinical “sentence”, an array of implicated “drugs”, and an array of specific adverse “reactions”.

**Figure S2.** Example of a clinical note annotation conforming to the standardized JSON schema. The figure presents a sample raw clinical text alongside its structured extraction. It illustrates how unstructured clinical narratives (e.g., patient reporting headache due to nicorandil) are accurately parsed into specific “sentence”, “drugs”, and “reactions” fields to build the standardized dataset.

**Figure S****3.** Prompt engineering framework for ADE recognition in Chinese. The schematic details three step-wise augmentation strategies for LLMs. (A) NAG serving as a zero-shot baseline. (B) SAG integrating static clinical diagnostic rules. (C) RAG dynamically injecting top-5 similar cases for context-aware, few-shot prompting. ADE: adverse drug event; LLM: large language model; NAG: none-augmented generation; RAG: retrieval-augmented generation; SAG: static-augmented generation.

**Figure S4.** Illustrative examples of L1, L2, and L3 recognition precision levels. These comparisons between annotated gold standards and identified outputs clarify the study’s entity-matching logic. L1 requires perfect alignment of all fields; L2 allows minor entity discrepancies within a correctly matched sentence; and L3 permits partial overlap when multiple adverse drug events are present.
